# Calorie restriction plugs lithocholic bile acid into the lysosomal AMPK pathway

**DOI:** 10.1093/lifemeta/loaf005

**Published:** 2025-02-11

**Authors:** Benoit Viollet

**Affiliations:** Université Paris Cité, CNRS, Inserm, U1016, Institut Cochin, 24 rue du faubourg Saint Jacques, Paris 75014, France

**The dietary intervention of calorie restriction (CR) is known to have the most profound effects on healthspan and lifespan across several species. In two recent back-to-back articles published in *Nature*, Qu *et al*. [1, 2] revealed that lithocholic acid (LCA), a singular secondary bile acid found to be elevated in the serum of calorie-restricted mice, is sufficient to mimic the effects of CR by delaying the onset of age-associated phenotypes in mice, and promoting lifespan extension in nematodes and flies. The authors showed that the anti-ageing effects of LCA are mediated through a conserved mechanism resulting in the activation of the lysosomal adenosine-monophosphate (AMP)-activated protein kinase (AMPK) pathway through the TUB-like protein 3 (TULP3)-sirtuin (SIRT)-vacuolar H** ^**+**^ **-ATPase (v-ATPase) axis to mediate the benefits of CR.**

It has long been postulated that nutritional interventions are reliable non-pharmacological therapies for promoting healthspan and extending lifespan across a wide range of species. The interrelationship between nutrition and longevity was first introduced in 1935 by McCay *et al.*, showing that the restriction of calories without malnutrition prolongs mean and maximal lifespan in rats compared with *ad libitum* feeding [3]. Calorie restriction (CR), defined as a reduction of caloric intake without compromising basic nutritional needs, remains the most consistent dietary intervention to effectively lengthen lifespan and delay the onset of age-related functional decline in yeast, worms, flies, rodents, and non-human primates [4, 5]. Data collected from observational and randomized clinical trials indicate that CR has similar beneficial effects on promoting healthspan and protecting against the development of health complications and malignancies associated with ageing in humans [6]. However, while CR greatly delays the onset of ageing-related diseases in humans, further studies will be necessary to elucidate its impact on promoting longevity in humans [6]. Dietary interventions (such as a reduction in calorie intake or intermittent fasting) are reputed to improve various health parameters associated with inflammation (e.g., age-related disorders associated with chronic low-grade inflammation) and have garnered particular interest as non-pharmacological therapeutic interventions to prevent or delay the onset of many chronic diseases, including type 2 diabetes, obesity, cardiometabolic diseases, various forms of cancer, neurodegeneration, autoimmune, and other inflammatory disorders [5, 6]. Therefore, the overall understanding of the mechanisms responsible for CR-induced delay in age-related functional decline has the potential to reveal targets for drugs and therapeutic strategies directed at promoting healthy ageing. In recent decades, identifying endogenous metabolites and developing compounds to mimic the physiological and functional responses to CR without dietary limitations, while providing long-term health benefits, has remained a significant challenge. In two recent back-to-back articles published in *Nature*, Qu *et al*. [1, 2] identified a CR-mediated upregulated circulating metabolite accounting for the rejuvenating benefits of CR in aged mice, as well as for the ability to extend lifespan in nematodes and fruit flies.

It is fascinating that a single nutritional intervention, CR, can provide broad-spectrum benefits for extending healthspan and lifespan across various species, from yeast to primates. Long-term CR is known to influence a variety of signalling pathways that control growth, metabolism, oxidative stress, damage repair, inflammation, and autophagy contributing to a slower physiological decline during ageing. Remarkably, most of the genes associated with increased lifespan, first identified in invertebrates, are part of key nutrient‐sensing pathways evolutionarily conserved in mammals involving adenosine-monophosphate (AMP)-activated protein kinase (AMPK), mammalian target of rapamycin (mTOR), sirtuins (SIRTs), nuclear factor erythroid 2-related factor 2 (NRF2), and insulin/insulin-like growth factor (IGF)/forkhead box O (FOXO) signalling [4, 5]. Hence, targeting these nutrient-sensing metabolic pathways to mimic caloric restriction-mediated benefits represents an interesting therapeutic strategy to reduce the incidence of several age-related diseases without enforcing long-term CR in patients. Several nutraceuticals and pharmaceuticals, including resveratrol and metformin, have been reported to act as calorie restriction mimetics (CRMs), as evidenced by their health-promoting and longevity-extending effects in various experimental model organisms [5]. Notably, most of these effects were shown to be dependent on AMPK signalling, an evolutionary conserved signalling pathway activated under CR [7]. AMPK has emerged as a key energy sensor with the ability to metabolically reprogram and adapt the cell to external cues. AMPK can be activated by a canonical (AMP/adenosine diphosphate (ADP)-dependent) pathway in response to cellular energy stress, or by non-canonical (AMP/ADP-independent) mechanisms involved in nutrient sensing or changes in intracellular Ca^2+^ levels [8]. Interestingly, the essential role of AMPK in the response to glucose starvation is conserved across eukaryotes. In mammalian cells, the mechanism involves the activation of a pool of AMPK localized at the surface of lysosome. Sensing of low glucose levels requires glycolysis and the production of fructose-1,6-bisphosphate (FBP), a substrate of the glycolytic enzyme aldolase. Detection of the lack of FBP by aldolase induces the inhibition of the aldolase/lysosomal proton pump vacuolar H^+^-ATPase (v-ATPase) complex, changing its interaction with the regulator-axin complex that tethers the liver kinase B1 (LKB1), an AMPK upstream kinase, to phosphorylate and activate lysosomal AMPK [8].

Following a CR intervention, metabolic adaptive processes are associated with changes in circulating metabolite profiles. In particular, correlations between serum metabolomic signatures and healthspan metrics have been reported. Using global mass spectrometry-based metabolomics approaches, Qu *et al*. identified a distinct metabolite-based signature in the serum of CR-treated versus non-CR-treated mice. A total of 1215 metabolites were altered after CR (approximately 70% of *ad libitum* food intake) over 4 months [1]. Interestingly, treatment with serum from CR-treated mice was sufficient to activate AMPK in primary hepatocytes and myocytes. Perfusion of the serum from CR-treated mice into *ad libitum*-fed mice resulted in the activation of AMPK in the liver and skeletal muscle. Building on these results, the authors focussed on searching for candidates capable of activating AMPK in cultured cells. Lithocholic acid (LCA) was the only metabolite able to activate AMPK at concentrations comparable to those detected in the serum of CR-treated mice. Various derivatives of LCA (iso-, 3-oxo-, allo-, 3-oxoallo-, and isoallo-LCA) were unable to activate AMPK or reproduce the healthspan- and lifespan-extending effects of LCA. LCA is a secondary bile acid formed in the large intestine as the result of dehydroxylation of the primary bile acid chenodeoxycholic acid (CDCA) by bacterial 7α-dehydroxylases. The rise of LCA during CR was attributable to the gut microbiome as the effect of CR on LCA levels was blunted in germ-free or antibiotic-treated mice. In addition, transplantation of faeces from CR-treated mice into germ-free or antibiotic-treated mice significantly increased the levels of LCA in the serum and faeces of transplanted mice. This model is consistent with previous studies showing that the beneficial effects of CR or CRMs on the ageing process are associated with modifications of the gut microbiome composition leading to changes in metabolite production [5]. Notably, healthy centenarians exhibit specific gut microbiota signatures with bacterial species capable of generating secondary bile acids leading to increased levels of LCA and its derivatives. However, much remains to be uncovered whether LCA is necessary or sufficient for mediating the effects of CR. It would be interesting to test the capacity of CR interventions to activate AMPK and improve age-related frailty in germ-free mice or antibiotic-treated mice, which are incompetent to increase LCA levels in response to CR.

Next, to elucidate the mechanism of LCA-induced AMPK activation, Qu *et al*. investigated the canonical and non-canonical routes leading to AMPK activation [2]. After ruling out the contribution of changes in AMP/ATP ratio or Ca^2+^ levels, the authors demonstrated that LCA activates AMPK through the lysosomal pathway. Mutation of aldolase to block AMPK activation in the lysosomal glucose-sensing pathway did not block LCA-mediated AMPK activation, suggesting that LCA acts downstream of aldolase to engage the lysosomal AMPK pathway. Exploring the post-translational modifications of v-ATPase in response to LCA treatment revealed that regulating its acetylation is crucial, as shown by the prevention of AMPK activation by LCA in the presence of deacetylase inhibitors. The authors demonstrated that the deacetylation by SIRTs of three acetylated lysine residues (K52, K99, and K191) on the V1E1 subunit of v-ATPase is the key mechanism for LCA-mediated AMPK activation through the lysosomal pathway. Notably, mutation of the lysine to glutamine, which mimics the constitutively acetylated V1E1, blocked AMPK activation by LCA and conversely, mutation of the lysine to arginine, which mimics the constitutively deacetylated V1E1, led to AMPK activation but without further activation by LCA. The levels of K99 acetylation were inversely correlated with the activation of AMPK in the skeletal muscle and liver from CR-treated mice or mice administrated LCA, as well as in cells incubated with LCA or serum from CR-treated mice. To elucidate the mechanism by which LCA activates SIRTs, cellular levels of nicotinamide adenine dinucleotide (NAD^+^), which drive the deacetylase activity of SIRT, were measured. Surprisingly, LCA activated SIRTs prior to the increase in NAD^+^ levels. Furthermore, LCA did not directly activate SIRTs in a cell-free system, suggesting that SIRT activation occurs through a cellular partner. Based on this hypothesis, the study then focussed on identifying TUB like protein 3 (TULP3) as a SIRT1-binding partner and a specific target for LCA, but not its derivatives. Binding of LCA to TULP3 allosterically activates SIRTs, stimulating the deacetylation of the v-ATPase V1E1 subunit, which in turn activates lysosomal AMPK via the lysosomal pathway (Fig. 1).

**Figure 1:**
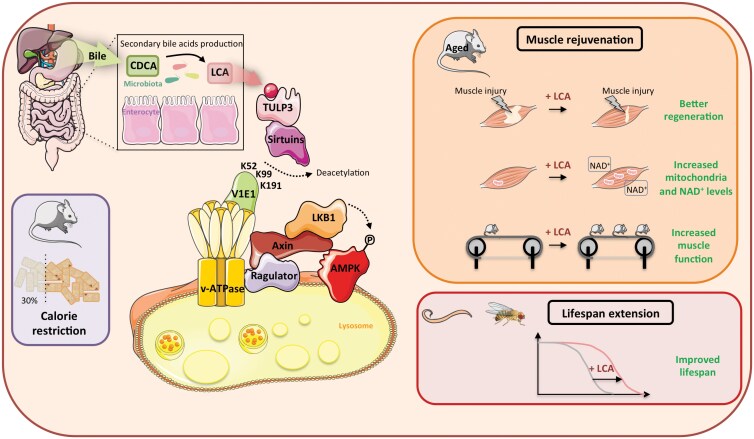
CR causes increased production of the secondary bile acid LCA from the bacterial dehydroxylation of the primary bile acid CDCA. LCA binds to the TULP3-sirtuin complex, stimulating deacetylation of the V1E1 subunit of the v-ATPase on K52, K99, and K191 residues, which in turn triggers the regulator-axin complex to recruit LKB1, the upstream kinase of AMPK, resulting in the phosphorylation and activation of lysosomal AMPK. LCA can recapitulate the anti-ageing effects of CR by inducing muscle rejuvenation in aged mice (enhanced muscle regeneration, increased number of mitochondria and NAD^+^ levels, and improved muscle function and performance) and promoting lifespan extension in nematodes and flies.

Notably, the administration of LCA to both male and female mice for 1 month triggered AMPK activation in skeletal muscle and replicated the beneficial effects of CR on muscle rejuvenation and performance in aged (1.5-year-old) mice (Fig. 1). Of note, the effects of LCA on *ad libitum*-fed mice were not associated with the loss of muscle mass, which is an adverse effect observed with CR due to a lack of amino acid supplementation. LCA treatment increased the number of oxidative muscle fibres, contributing to the preservation of muscle force and endurance. For instance, LCA increased mitochondrial content and NAD^+^ levels in the muscle, both of which are downstream consequences of AMPK activation. LCA-stimulated production of NAD^+^ could create a positive feedback loop on NAD^+^-activated SIRTs, enhancing their activity to promote health benefits. To further explore the implication of AMPK signalling in mediating the therapeutic effects of LCA, the authors used muscle-specific *Ampk*-knockout mice. The improvements in muscle function by LCA administration were fully abrogated in the absence of muscle AMPK. However, a direct interpretation of these findings may be confounded by the exercise intolerance of muscle-specific *Ampk*-knockout mice. Reduced muscle mitochondrial content and neuromuscular alterations in this model could also contribute to the resistance to the effect of LCA on skeletal muscle remodelling and performance. The authors concluded that AMPK activation is necessary for the muscle rejuvenating effects of LCA, in line with the observation of elevated muscle mitochondrial abundance and enhanced contractile performance in AMPK gain-of-function mice or aged mice treated with the AMPK activator aldometanib, targeting the lysosomal pathway [9]. To support the role of the TULP3-SIRT-v-ATPase axis in LCA action, muscle-specific expression of a mutant mimicking the deacetylated state of the v-ATPase V1E1 subunit, which led to AMPK activation, was sufficient to improve muscle function of aged mice. All these beneficial effects were lost upon deletion of muscle AMPK. Additionally, mutation of Y193, P195, K333, and P336 residues of TULP3, forming the binding pocket for LCA, blocked LCA-mediated activation of SIRTs and AMPK. Expression of this mutant in the skeletal muscle abolished the rejuvenating effects of CR. Collectively, these findings indicate that LCA can mimic the effects of CR on muscle rejuvenation through the TULP3-SIRT1-v-ATPase-AMPK axis.

Previous studies have reported that LCA can extend the lifespan of yeast (*Saccharomyces cerevisiae*) and fruit fly (*Drosophila melanogaster*). The authors extend these findings by showing that LCA also significantly prolongs lifespan as well as healthspan in both *D. melanogaster* and the nematode *Caenorhabditis elegans* (Fig. 1). Deletion of AMPK abolished the anti-ageing effects of LCA, consistent with previous studies showing that AMPK mediates longevity in flies and nematodes induced by CR or CRMs. Knockout of SIRTs, the *C. elegans* TULP3 homologue *tub-1*, or the *D. melanogaster* homologue *ktub*, as well as mutations in residues involved in LCA binding in TULP3 homologues, all abolished LCA-mediated AMPK activation and lifespan extension. On the opposite, the expression of a deacetylated mutant of V1E1 was able to extend lifespan independently of LCA. These results indicate that the TULP3-SIRT-v-ATPase-AMPK axis mediates the effect of LCA on lifespan extension in worms and flies. Remarkably, re-expression of TULP3 in *tub-1* knockout worms and *ktub* knockout flies restored the effects of LCA on lifespan extension, indicating highly conserved signalling despite the lack of endogenous LCA in nematodes and flies.

In conclusion, these tour-de-force studies highlighted the importance of the v-ATPase-mediated AMPK activation in the mechanisms underlying the benefits of CR on healthspan and lifespan across the evolutionary scale. This is consistent with previous studies showing that metformin and aldometanib target the lysosomal pathway to extend healthspan and lifespan in worms [9, 10]. Qu *et al*. have identified a conserved TULP3-SIRT1-v-ATPase axis that, under CR, activates the lysosomal pool of AMPK to promote muscle rejuvenation in mice and healthspan and lifespan in worms and flies [1, 2]. They also provide evidence that the benefits of CR can be mimicked by the administration of LCA, an endogenous metabolite produced by the intestinal flora. These results suggest the importance of maintaining a healthy microbial ecology to support healthy ageing. These findings have also exciting therapeutic implications. Identifying LCA as a mediator of CR effects and its action through the TULP3-SIRT1-v-ATPase axis could pave the way for developing novel pharmacological or nutritional interventions to slow or reverse functional decline in elderly individuals. However, some important questions remain. For example, are there other natural metabolites induced by CR that activate SIRT or the lysosomal AMPK pathways? Is LCA administration sufficient to activate the lysosomal AMPK pathway in organs beyond the skeletal muscle and liver? What are its physiological impacts on healthspan? Further studies may also be useful in delineating the pathways downstream of lysosomal AMPK or SIRT activation that could lead to the positive changes induced by LCA in health and ageing. For instance, lysosomal AMPK activation has been shown to contribute to the anti-ageing effects of CR by inducing glutaminolysis and mitohormesis through the AMPK-PDZ domain-containing 8 (PDZD8)-glutaminase 1 (GLS1) axis in mice and worms [11]. In addition, skeletal muscle AMPK-mediated autophagy also plays an essential role in maintaining muscle integrity during ageing [12]. Lastly, it would be valuable to investigate further whether LCA has the potential to extend lifespan in mice when administered at different concentrations and ages, as observed in aged (1-year-old) mice treated with aldometanib, a lysosomal AMPK activator [9]. This is a critical area of inquiry, as recent studies reported a divergence in the mechanisms underlying the benefits of CR for improving healthspan or extending lifespan in mice, highlighting the complexity of the response to dietary interventions [13].

## References

[1] Qu Q , Chen Y, Wang Y et al Nature 2024. https://doi.org/10.1038/s41586-024-08329-5

[2] Qu Q , Chen Y, Wang Y et al Nature 2024. https://doi.org/10.1038/s41586-024-08348-2

[3] McCay CM , Crowell MF, Maynard LA. J Nutr 1935;10:63–79. 2520283

[4] Fontana L , Partridge L, Longo VD. Science 2010;328:321–6. 20395504 10.1126/science.1172539 PMC3607354

[5] Green CL , Lamming DW, Fontana L. Nat Rev Mol Cell Biol 2022;23:56–73. 34518687 10.1038/s41580-021-00411-4 PMC8692439

[6] Kebbe M , Sparks JR, Flanagan EW et al Expert Rev Endocrinol Metab 2021;16:95–108. 33957841 10.1080/17446651.2021.1922077 PMC9052419

[7] Burkewitz K , Zhang Y, Mair WB. Cell Metab 2014;20:10–25. 24726383 10.1016/j.cmet.2014.03.002 PMC4287273

[8] Steinberg GR , Hardie DG. Nat Rev Mol Cell Biol 2023;24:255–72. 36316383 10.1038/s41580-022-00547-x

[9] Zhang CS , Li M, Wang Y et al Nat Metab 2022;4:1369–401. 36217034 10.1038/s42255-022-00640-7 PMC9584815

[10] Ma T , Tian X, Zhang B et al Nature 2022;603:159–65. 35197629 10.1038/s41586-022-04431-8 PMC8891018

[11] Li M , Wang Y, Wei X et al Cell Res 2024;34:806–9. 39300254 10.1038/s41422-024-01021-3 PMC11528062

[12] Bujak AL , Crane JD, Lally JS et al Cell Metab 2015;21:883–90. 26039451 10.1016/j.cmet.2015.05.016 PMC5233441

[13] Di Francesco A , Deighan AG, Litichevskiy L et al Nature 2024;634:684–92. 39385029 10.1038/s41586-024-08026-3 PMC11485257

